# Novel Strategy for Non-Targeted Isotope-Assisted Metabolomics by Means of Metabolic Turnover and Multivariate Analysis

**DOI:** 10.3390/metabo4030722

**Published:** 2014-08-25

**Authors:** Yasumune Nakayama, Yoshihiro Tamada, Hiroshi Tsugawa, Takeshi Bamba, Eiichiro Fukusaki

**Affiliations:** 1 Department of Biotechnology, Graduate School of Engineering, Osaka University, 2-1 Yamadaoka, Suita, Osaka 565-0871, Japan; E-Mails: yasumune_nakayama@bio.eng.osaka-u.ac.jp (Y.N.); yoshihiro-tamada@hakutsuru.co.jp (Y.T.); hiroshi.tsugawa@riken.jp (H.T.); bamba@bio.eng.osaka-u.ac.jp (T.B.); 2 RIKEN Center for Sustainable Resource Science, 1-7-22 Suehiro-cho, Tsurumi-ku, Yokohama, Kanagawa 230-0045, Japan

**Keywords:** metabolomics, stable isotope, isotope-assisted metabolomics, metabolic turnover analysis, non-targeted analysis, gas chromatography, mass spectrometry, *Saccharomyces cerevisiae*

## Abstract

Isotope-labeling is a useful technique for understanding cellular metabolism. Recent advances in metabolomics have extended the capability of isotope-assisted studies to reveal global metabolism. For instance, isotope-assisted metabolomics technology has enabled the mapping of a global metabolic network, estimation of flux at branch points of metabolic pathways, and assignment of elemental formulas to unknown metabolites. Furthermore, some data processing tools have been developed to apply these techniques to a non-targeted approach, which plays an important role in revealing unknown or unexpected metabolism. However, data collection and integration strategies for non-targeted isotope-assisted metabolomics have not been established. Therefore, a systematic approach is proposed to elucidate metabolic dynamics without targeting pathways by means of time-resolved isotope tracking, *i.e.*, “metabolic turnover analysis”, as well as multivariate analysis. We applied this approach to study the metabolic dynamics in amino acid perturbation of *Saccharomyces cerevisiae*. In metabolic turnover analysis, 69 peaks including 35 unidentified peaks were investigated. Multivariate analysis of metabolic turnover successfully detected a pathway known to be inhibited by amino acid perturbation. In addition, our strategy enabled identification of unknown peaks putatively related to the perturbation.

## 1. Introduction

Isotope-labeling experimentation is a useful tool for understanding cellular metabolism. Tracking the fate of isotope-labeled substrates has played an important role in determination of metabolic pathways such as the Calvin-Benson cycle [1] and the Entner-Doudoroff pathway [2]. Furthermore, isotope-tracking experiments have been performed to elucidate the reverse reaction flow of known pathways including the reductive tricarboxylic acid cycle (TCA) cycle [3] and gluconeogenesis [4]. In these experiments, a limited number of metabolites was observed in the process of discovering a particular pathway.

Recent advances in metabolomics enabled monitoring of a huge number of metabolites with high reproducibility [5]. Metabolomics technology extends the applicability of isotope-assisted experiments to a global scale to develop “isotope-assisted metabolomics” [6], which can be classified into three groups: (i) elucidation of existing pathways on a global scale by detection of isotope-labeled substrates [7,8,9]; (ii) assignment of elemental formulas to all unknown metabolites by comparing isotopically labeled and non-labeled samples [10]; and (iii) estimation of the flux distribution at branch points of several metabolic pathways from the isotopic patterns of metabolites [7,11,12,13]. Furthermore, some data processing tools have been developed for non-targeted isotope-assisted metabolomics such as mzMatch-ISO, NTFD, and X^13^CMS [12,14,15]. Since metabolic pathways are still being updated even in the central metabolism of model organisms [16,17,18], the development of non-targeted approaches for isotope-assisted metabolomics is important. However, data collection and integration strategies of non-targeted isotope-assisted metabolomics to reveal unknown or unexpected metabolic pathways and intermediates have not been well developed.

Recently, Huang *et al.* introduced non-targeted isotope-assisted metabolomics to elucidate perturbation-related pathways using X^13^CMS [14]. This concept was advanced for studying metabolic dynamics because systematic non-targeted approaches for revealing perturbation-related metabolism had not been proposed. However, this study compared the isotopomers between samples at only a single time point. Since the isotope labeling speed varies according to the pathway [19], snapshot analysis is not sufficient to elucidate perturbation-related pathways on a global scale. Although time resolved labeling experiments, *i.e.*, “metabolic turnover analysis,” could overcome this problem [20,21,22], the data analysis strategy for metabolic turnover analysis has not yet been established. Therefore, a method for integration of both non-targeted turnover analysis and multivariate analysis is needed to facilitate the use of isotope-assisted metabolomics to increase understanding of biological processes.

**Figure 1:**
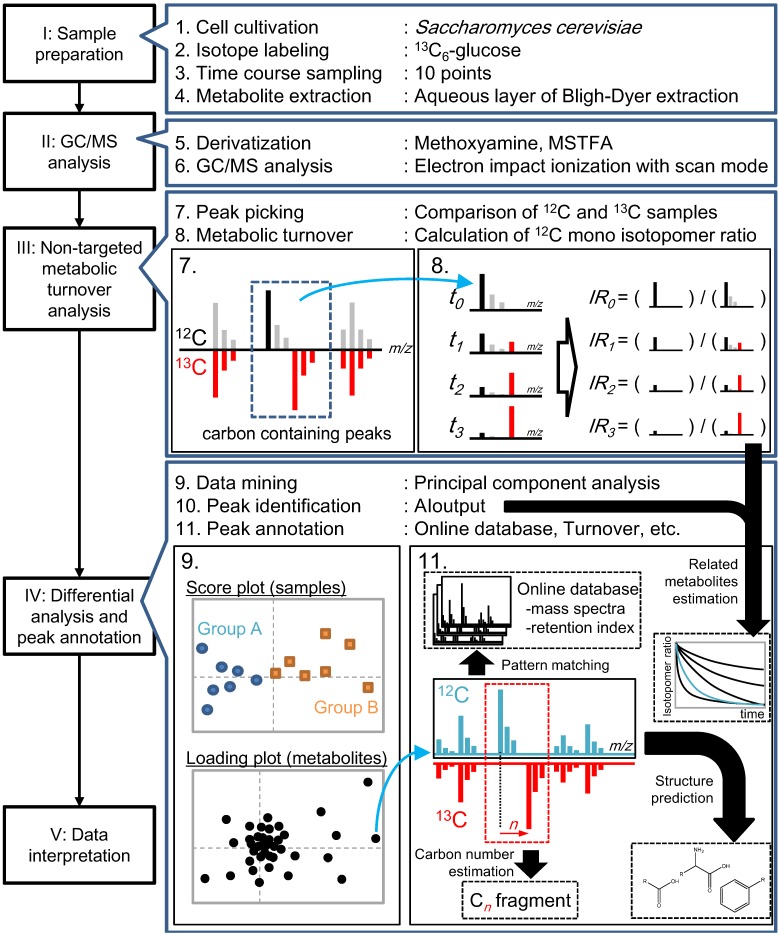
Non-targeted metabolic turnover analysis-based differential analysis of metabolic dynamics. Boxes on the left indicate the workflow of gas chromatography/electron ionization/mass spectrometry (GC/EI/MS)-based non-targeted metabolic turnover analysis. Procedural details are described in the boxes to the right.

Here we suggest a systematic approach for elucidation of the unknown and unexpected pathways related to perturbation by means of non-targeted metabolic turnover analysis and multivariate analysis (Figure 1). This approach highlights perturbation-related metabolites from a complicated dataset containing metabolites with different labeling speeds, which assisted in the elucidation of metabolic changes on a global scale. Gas chromatography coupled with electron impact mass spectrometry (GC/EI/MS) was utilized in this study for its well-established peak identification system [23], huge databases of fragment spectra [24,25], and software for structure prediction [23,26]. To demonstrate the potential of our approach, we investigated pathways related to amino acid perturbation in *Saccharomyces cerevisiae*.

## 2. Results and Discussion

### 2.1. Strategy Overview

The scheme for non-targeted metabolic turnover analysis is shown in Figure 1. Sample preparation and the gas chromatography/mass spectrometry (GC/MS) method for isotope tracing of metabolites are outlined in Processes I and II, respectively. In metabolic turnover analysis, time course sampling was performed to track the fate of isotope labels. Non-labeled and ^13^C-labeled samples were also prepared to facilitate peak picking. All detectable metabolites were monitored using scan-mode analysis on a mass spectrometer. Processes III and IV present the methods for data analysis including peak picking, isotope ratio calculation, and multivariate analysis. In Process III, the peaks containing carbon atoms are selected from the data set of both non-labeled and ^13^C-labeled samples. Then, the metabolic turnover of each peak was calculated. In Process IV, differential analysis was performed to elucidate perturbation-related pathways and rate the priority of the peaks. High priority peaks were annotated based on several aspects. Finally, the effect of biological perturbation is discussed in Process V.

### 2.2. Non-Targeted Metabolic Turnover Analysis

In order to perform non-targeted metabolic turnover analysis (Figure 1-III), the peak picking method for collecting the carbon-containing mass fragment information derived from yeast was required. Therefore, a comparative approach for mass spectra between non-labeled and ^13^C-labeled samples was used (Figure 1(1-7)). The non-labeled and ^13^C-labeled metabolites were extracted from the X2180 strain cultivated under minimal synthetic medium with natural isotopic glucose and ^13^C_6_-glucose, respectively. We identified 69 peaks with fragment ions containing carbon atoms (Table 1).

Based on these peaks, non-targeted metabolic turnover analysis (Figure 1(1-8)) was performed on three samples: X2180 strain grown under minimal synthetic medium and X2180 and BY4742 strains grown under minimal synthetic medium with amino acid supplement. For the turnover analysis, 60 out of the 69 peaks were monitored while the remaining nine peaks (Peak-22, -35, -37, -41, -42, -44, -56, -58, and -65) were not observed consistently, probably due to a small metabolite pool. The isotopomer ratio (*IR*) of each metabolite was calculated using the following equation:

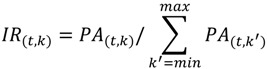
(1)
where *PA* represents peak area, *t* represents each sampling time, *k* represents the *m*/*z* of a fragment of the metabolite, *min* represents the *m*/*z* of ^12^C-fragments, and *max* represents ^13^C-fragments of the metabolite described on Table 1. The peak area used in this equation was from the *m*/*z* of the ^12^C-monoisotope to the ^13^C-monoisotope of a fragment ion. Only the *IR*_(*t,min*)_ was used for further metabolic turnover analysis. In order to reduce the effect of the natural abundance of an isotopomer, the metabolic turnover was divided by the maximum *IR* of each metabolite.

**Table 1:** Carbon-containing peaks derived from *S. cerevisiae*.

Peak No.	RT * (s)	RI **	Fragment (*m*/*z*)		Automatically Identified Name ***
			^12^C	^13^C	
Peak-01	222.0	-	171	172	
Peak-02	282.3	1043.8	174	177	Pyruvate + Oxalacetic acid::C00022 + C00036
Peak-03	318.6	1094.2	116	118	Alanine_2TMS::C00041
Peak-04	332.3	1115.3	102	103	Glycine_2TMS::C00037
Peak-05	365.0	1165.8	130	133	2-Aminobutyric acid::C02261
Peak-06	393.0	1207.3	144	148	Valine_2TMS::C00183
Peak-07	420.0	1252.8	116	118	Serine_2TMS::C00065
Peak-08	441.0	1286.2	158	163	Isoleucine_2TMS::C00407
Peak-09	442.6	1288.6	117	119	Threonine_2TMS::C00188
Peak-10	446.4	1294.5	142	146	Proline_2TMS::C00148
Peak-11	449.4	1299.1	174	175	Glycine_3TMS::C00037
Peak-12	453.3	1306.2	247	251	Succinic acid (or aldehyde)::C00042
Peak-13	474.0	1343.8	245	249	Fumaric acid::C00122
Peak-14	477.0	1349.2	204	206	Serine_3TMS::C00065
Peak-15	480.0	1354.4	188	190	Alanine_3TMS::C00041
Peak-16	491.4	1374.2	218	221	Threonine_3TMS::C00188
Peak-17	516.0	1418.5	160	163	
Peak-18	526.2	1438.3	218	221	Homoserine_3TMS::C00263
Peak-19	538.8	1462.1	232	234	
Peak-20	546.0	1475.6	233	236	Malic acid::C00149
Peak-21	550.8	1484.4	188	193	
Peak-22	558.0	1497.5	112	118	
Peak-23	562.8	1507.2	232	235	Aspartic acid_3TMS::C00049
Peak-24	565.8	1513.5	176	180	Methionine_2TMS::C00073
Peak-25	568.8	1519.7	156	160	Pyroglutamic acid::C01879
Peak-26	571.8	1525.9	174	178	4-Aminobutyric acid::C00334
Peak-27	574.2	1530.8	155	159	
Peak-28	576.6	1535.8	227	231	
Peak-29	589.2	1561.3	247	251	
Peak-30	591.0	1564.8	275	281	
Peak-31	600.6	1583.9	227	231	
Peak-32	609.0	1600.3	142	146	
Peak-33	612.0	1606.9	246	250	Glutamic acid_3TMS::C00302
Peak-34	619.8	1624.1	192	200	Phenylalanine_2TMS::C00079
Peak-35	628.6	1643.1	116	118	
Peak-36	631.8	1650.0	275	279	
Peak-37	634.3	1655.3	234	238	
Peak-38	636.6	1660.3	116	118	Asparagine_3TMS::C00152
Peak-39	639.0	1665.4	290	293	
Peak-40	647.4	1683.0	275	279	
Peak-41	653.7	1696.2	173	177	
Peak-42	661.7	1714.4	227	231	
Peak-43	664.4	1720.4	205	207	
Peak-44	667.0	1726.5	274	280	
Peak-45	672.0	1737.9	231	235	
Peak-46	677.7	1750.8	217	220	
Peak-47	683.4	1763.6	156	160	Glutamine_3TMS::C00064
Peak-48	699.6	1799.5	273	278	Citric acid + Isocitric acid::C00158+C00311
Peak-49	701.4	1803.8	142	146	Ornithine::C00077
Peak-50	706.8	1817.0	117	119	
Peak-51	719.7	1847.9	174	175	Lysine_3TMS::C00047
Peak-52	723.6	1857.2	319	323	Allose_1_Major::C01487
Peak-53	737.1	1888.9	205	207	
Peak-54	740.4	1896.5	319	323	Glucose_2_Minor::C00031
Peak-55	745.2	1908.5	174	175	Lysine_4TMS::C00047
Peak-56	747.5	1914.3	319	323	
Peak-57	749.4	1919.2	254	259	Histidine_3TMS::C00135
Peak-58	753.8	1930.3	218	220	
Peak-59	757.7	1940.3	217	220	
Peak-60	762.6	1952.5	204	206	
Peak-61	782.9	2003.1	204	206	
Peak-62	807.8	2068.6	204	206	
Peak-63	837.0	2147.1	326	331	
Peak-64	871.7	2243.7	144	148	
Peak-65	981.4	2578.0	217	220	
Peak-66	1015.6	2691.5	204	206	
Peak-67	1021.5	2711.8	361	367	Trehalose::C01083
Peak-68	1057.4	2837.7	361	367	Melibiose_1_Major::C05402
Peak-69	1074.6	2899.1	204	206	

* Retention time (RT);** Retention index (RI) was calculated in the range from 1000 to 4000; *** Peak identification was performed using AIoutput.

**Figure 2:**
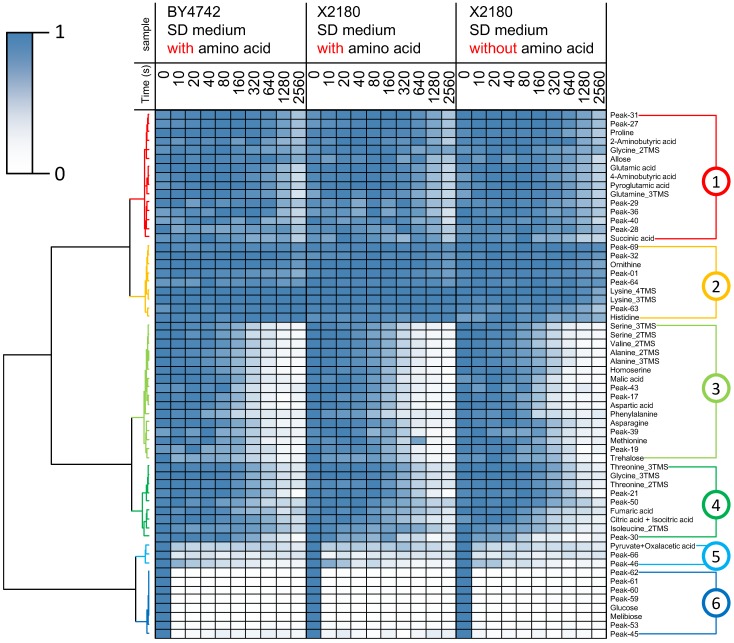
Metabolic turnover of detected peaks. The heat map indicates the ratio of the ^12^C-monoisotopic mass at each sampling time. The color gradation is indicated at the upper left. The data are standardized by the maximum value of each isotopomer. Peak clusters were calculated by hierarchical cluster analysis (HCA) using Euclidian distances and Ward’s method linkage criteria.

The *IR*s of 60 metabolites are shown in Figure 2. Hierarchical cluster analysis (HCA) was used to group the metabolic turnovers of the peaks into six clusters. Clusters 1 and 2 appeared to not reach the isotopic steady state within the time limit, and Cluster 2 was almost not labeled. Clusters 4 and 5 were slowly labeled and almost reached the isotopic steady state. Clusters 3 and 6 were rapidly labeled.

### 2.3. Differential Analysis and Peak Annotation

Differential analysis (Figure 1(1–9)) was performed to select important peaks related to amino acid perturbation. The metabolic turnover of detected peaks was similar in all samples (Figure 2). Therefore, principal component analysis (PCA) was applied based on the difference in isotopomer ratios to mine fluctuated pathways. The differential isotopomer ratio (*DIR*) of each metabolite was calculated by subtracting the average at each time point as follows:

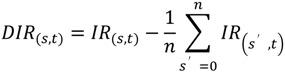
(2)
where *s* represents each sample, *t* represents the sampling time, and *n* represents the sample number including all sample types at time *t*. The *DIR* was then applied to PCA, taking every sampling time for each sample as an independent class and *DIR* as the element. Since the differences among the *DIR*s of the samples were important, preprocessing was not performed.

Statistical tests provide alternative analyses with the following considerations: (i) the sampling point; (ii) the sampling number; and (iii) the analysis of successive data. The two-sample *t*-test can be applied to analyze several replicates; however, the appropriate time point for differential analysis is difficult to estimate since the isotopically non-stationary phase depends on the metabolites, labeling sources, conditions, and organisms employed. In addition, a *t*-test of successive data requires a data integration process for effective detection and an estimation of appropriate data points to avoid multiple comparison problems. The Friedman test is another alternative for the analysis of successive data. However, its sensitivity also depends on the sampling time and number because the turnover data has a nonparametric and standardized data structure. Particularly, the sampling number of the isotopically stationary phase probably affects to the detection power because the *DIR* approaches zero at those points. To avoid these problems, we used PCA in this study.

**Figure 3:**
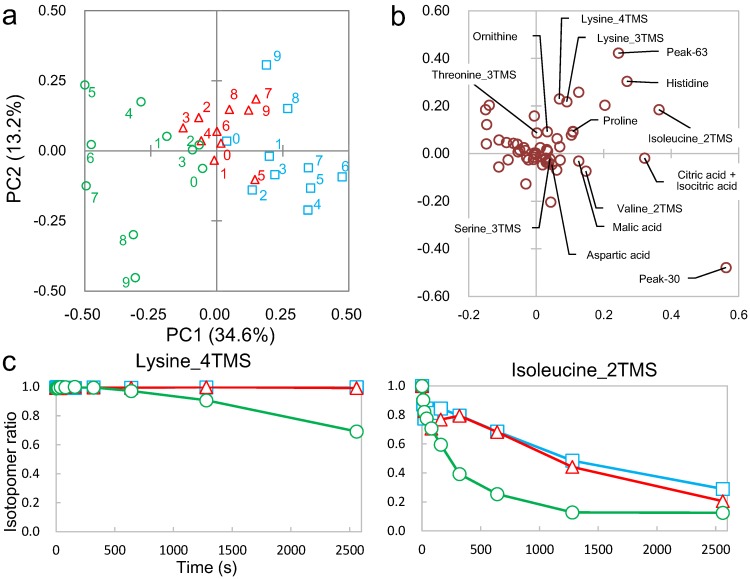
Principle component analysis (PCA) of metabolic turnover differences. (**a**) PCA score plot of each sampling time of three respective sample types: BY4742 under SD medium with amino acid supplement (open square), X2180 under SD medium with amino acid supplement (open triangle), and X2180 under SD medium without amino acid supplement (open circle). Numbers 0–9 indicate sampling times: 0, 10, 20, 40, 80, 160, 320, 640, 1280, and 2560 s, respectively. The horizontal axis indicates principal component (PC) 1, and the vertical axis indicates PC 2. The values in parentheses are the percent variances of each PC; (**b**) loading plot of peaks used in PCA. The labeled peaks represent metabolites that are significantly correlated with the PCs (Table S1). Axes are labeled as in (**a**); (**c**) Time courses of isotopomer ratios of ^12^C-monoisotopic lysine and isoleucine peaks. The horizontal axis indicates the sampling time, and the vertical axis indicates the isotopomer ratios. Sample type symbols are the same as for (**a**).

PCA projects the data to principal components defined by the magnitude of variance. The first principal component was calculated to maximize sample differences based on *DIR* variance, and the second was calculated to orthogonally maximize the sample differences to the first coordinate. Since the *DIR* approaches zero at early time points and at late, isotopically stationary time points, isotopically non-stationary time points are scattered from the origin on the score plot (Figure 3a). Moreover, the *DIR*s of metabolites on less perturbed pathways approach zero and are clustered around the origin on the loading plot. In contrast, the *DIR*s of metabolites on perturbed pathways maintain high values over time and are separated from the origin*.*

Candidate metabolites from fluctuated pathways were selected using the PCA loading plot (Figure 3b). Recently, Yamamoto *et al.* reported a statistical procedure for selecting metabolites using factor loadings [27]; separation of the score plot by sample types and sampling times herein permitted use of this criterion for metabolite selection. As detailed previously, variables significantly correlated with the principal components were calculated as follows:

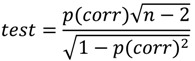
(3)
where *p*(*corr*) and *n* represent the factor loading and the sample number, respectively. The test statistic, *test*, has a *t*-distribution with (*n* − 2) degrees of freedom.

The resulting PCA score plot displays the different sample types and sampling times (Figure 3a), and the PCA loading plot indicates the peaks that contribute to the difference (Figure 3b). Different sample types showed distinct profiles over the time course from 80 to 2560 s. The peaks that contributed to the separation were selected according to Equation (3) (Table S1). To elucidate the negative effect of the amino acid supplement, peaks that positively correlated to PC1 and/or PC2 were selected for further analysis (labeled peaks in Figure 3b). The largest difference in isotopomer ratio among samples occurred at different times for different metabolites (Figure 3c). This result demonstrates the importance of metabolic turnover analysis to study global metabolism compared with snapshot analysis.

The selected peaks were identified automatically (Figure 1(1–10)) using a data processing tool [23] and our in-house library, and the identifications of 34 out of 69 peaks were manually verified by comparison with the library spectra (Table 1). Of the remaining 35 unidentified peaks, only two (Peak-30 and Peak-63) positively correlated with the separation (Figure 3b).

**Figure 4:**
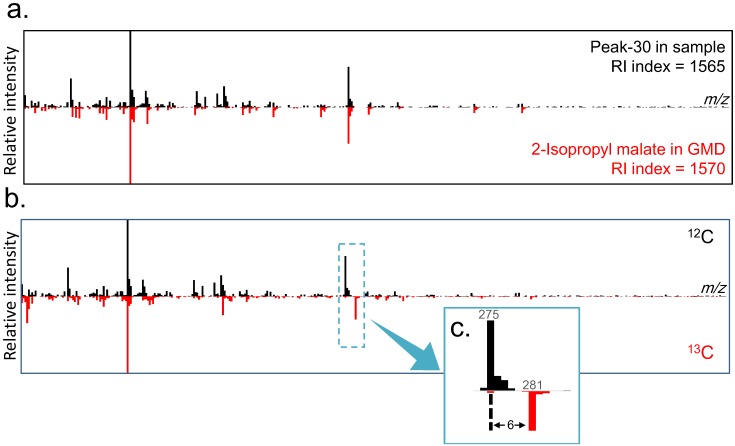
Mass spectra of Peak-30. (**a**) Mass spectra of Peak-30 in the sample (**upper**) and 2-isopropyl malate in the Golm Metabolome Database (**lower**); (**b**) Mass spectra of ^12^C- (upper) and ^13^C-labeled (lower) Peak-30; (**c**) Magnification of the boxed section in (**b**). Gray numbers indicate the *m*/*z* values.

**Figure 5:**
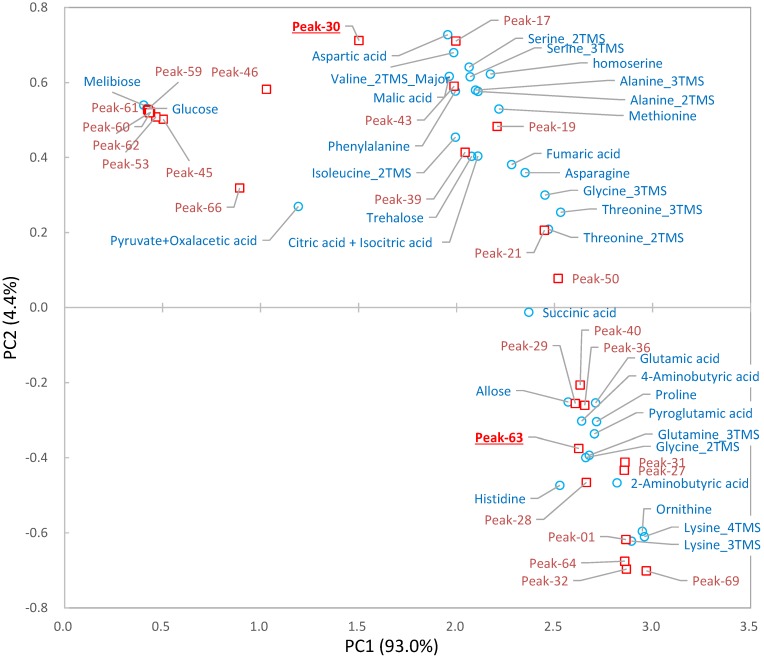
Score plot for principal component analysis (PCA) of metabolic turnover in *S. cerevisiae* X2180. The horizontal axis indicates principal component (PC) 1, and the vertical axis indicates PC 2. The values in parentheses are the percent variances of each PC. Open circles, and open squares indicate identified and unidentified peaks, respectively. Growth conditions: SD medium without amino acids.

These two peaks were characterized using several techniques including an online database search, carbon number determination, substructure prediction, and metabolic distance estimation (Figure 1(1–11)). From this point onwards, Peak-30 will be used as an example to describe the different aspects of peak annotation. To search the online database, the spectrum was uploaded to the Golm Metabolome Database (GMD) [24], yielding 2-isopropyl malate and 2-oxoglutarate as candidates (Table S2). Whereas most criteria were similar for both candidates, the dot-product match score (1-dotproduct) was higher for 2-isopropyl malate (Table S2, Figure 4a). Next, Peak-30 was estimated to have more than six carbon atoms, which was determined from the maximum difference (6) of the ^12^C and ^13^C isotopomers of the detected fragments (Figure 4b,c). Since 2-isopropyl malate and 2-oxoglutarate are composed of seven and five carbons, respectively, this makes 2-oxoglutarate an unlikely candidate. Nineteen substructures were predicted by a decision tree on GMD [26] with more than 90% probability, all of which completely matched the substructures of 2-isopropyl malate (Table S3). In addition, PCA was performed on the metabolic turnover data of X2180 in order to visualize the metabolic distance (Figure 5), which is an indicator of the distance between glucose and metabolites on the metabolic map [28]. On the PCA score plot, Peak-30 is located closer to glucose than the amino acids or most TCA cycle metabolites, indicating that Peak-30 is probably a metabolite near the central metabolism and not a derivation of TCA cycle metabolites. Together, these results led to annotation of Peak-30 as 2-isopropyl malate. This annotation was further tested by spiking in an authentic standard during analyses (Figure S2a), which confirmed the level 1 identification of Peak-30 as 2-isopropyl malate [29].

Peak-63 was determined to contain more than six carbon atoms, as the maximum carbon number of the detected fragments was six (Figure S1b). The spectrum did not match any metabolites in GMD within the threshold (Table S2); however, substructure prediction by the decision tree indicated that Peak-63 could be an amino acid containing an aromatic functional group (Table S4). Furthermore, turnover of Peak-63 was relatively slow and similar to histidine (Figure 3b and Figure 5). From these findings, Peak-63 was inferred to be a compound related to histidine metabolism. The spectrum of the corresponding derivative of histidine, histidine-4TMS, was found in GMD. The spectral search did not select histidine-4TMS as a candidate because there were considerable mismatches in the MS scan range between the GMD and our experiment. The highest intensity of histidine-4TMS, which occurs at 73 *m*/*z* in GMD, is out of our scan range and lowered the dot-product match score of the spectra (Figure S1a). Further testing of this annotation was conducted by spiking the authentic standard (Figure S2b), which confirmed the level 1 identification of Peak-63 as a histidine derivative [29].

### 2.4. Biological Discussion from the Results

**Figure 6:**
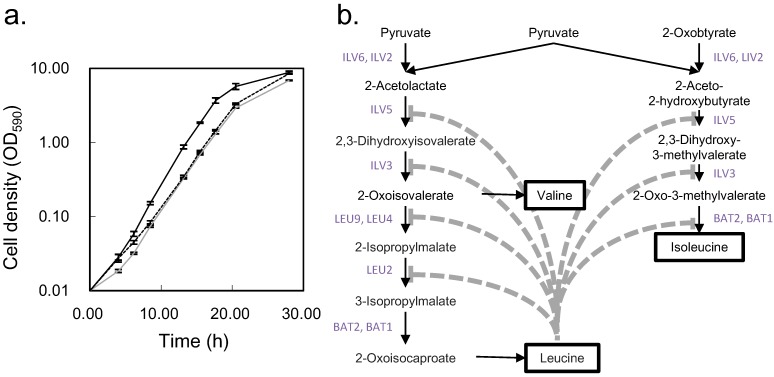
Growth curve and metabolic pathway of branched chain amino acid biosynthesis of *S. cerevisiae*. (**a**) Growth curve of each sample. The horizontal axis indicates the sampling time, and the vertical axis indicates the cell density on a logarithmic scale. The black, dotted, and gray lines indicate BY4742 under synthetic dextrose (SD) medium with amino acid supplement, X2180 under SD medium with amino acid supplement, and X2180 under SD medium without amino acid supplement, respectively; (**b**) Metabolic pathway of valine, leucine, and isoleucine in *S. cerevisiae* (29). Capital letters indicate the corresponding enzymes for the reactions. Dotted lines indicate feedback inhibition by leucine.

The *S. cerevisiae* BY4742 strain is often used as a parental strain, such as in the single gene knockout collection of the European *S. cerevisiae* archive for functional analysis (EUROSCARF) [30]. The BY4742 strain grows slowly under minimal medium compared to the reference strain X2180 (Figure 6a). However, the growth of X2180 is also slow after addition of supplemental nutrition (leucine, lysine, histidine, and uracil), which is essential for BY4742. Therefore, we investigated the effect of these supplements on metabolism using non-targeted metabolic turnover analysis. On the differential analysis score plot (Figure 3a), samples taken between 160 to 2560 s were separated by nutritional condition. In addition, the loading plot (Figure 3b) indicated that the greatest contributors to the separation belonged to the branched chain amino acid pathway or the TCA cycle. Whereas metabolic turnover of most contributing metabolites was slowed by supplemental nutrition, lysine and histidine were not labeled within 2560 s (Figure 1 and Figure 3c), indicating that these amino acids might not be synthesized in this condition.

Leucine is known to inhibit many reactions on the branched chain amino acid pathway [31], and our results may reflect this phenomenon. Additionally, previous research demonstrated that the growth of X2180 cells decreased with the addition of leucine but was recovered by adding valine and isoleucine [32]. The same study also showed that addition of both histidine and lysine maintained growth, while the presence of only one of the two retarded growth. Therefore, it is likely that the main reason for slow growth of BY4742 is feedback inhibition by leucine. Our result also indicated that the TCA cycle was affected by the amino acid supplement. However, the reason for and the effect of the slow turnover of TCA cycle intermediates require further investigation.

Time courses of isotopomer ratios were analyzed in this study. On the other hand, the labeling patterns at isotopically stationary phase are often used to elucidate metabolic dynamics. The labeling pattern provides information about branches and confluences on the metabolic map. Therefore, the patterns are used to calculate the flux in central metabolic pathways, specifically glycolysis, the pentose phosphate pathway, and the TCA cycle [6,13]. However, the approach does not extend well to more peripheral pathways, including amino acid synthesis, probably because fewer branches and confluences are present. Furthermore, initial information about the metabolic pathway is necessary to elucidate the dynamics. In contrast, time course data provide the dynamics of specific metabolites without prior knowledge of their metabolic pathways. The flux or kinetics of the pathway also can be analyzed with additional information [19,33,34]. In the present study, unknown peaks were analyzed without a metabolic map; therefore, time course monitoring was necessary.

In this experiment, 69 peaks were detected as carbon containing peaks. In spite of non-target analysis, less than a hundred peaks were observed. This is probably caused by three reasons. Firstly, the intensity of the metabolites is not sufficient for the analysis. Since the metabolite abundance of *S. cerevisiae* is relatively low under minimum medium, the detected peaks are limited [35]; Secondly, some metabolites do not provide any carbon containing peaks. Sometimes, the highest fragment of a compound is derived from derivatization and no or low fragments derived from the compound are observed. Thirdly, the peak detection strategy is not fully developed. The problems will be solved by development of analytical system including sample preparation, analytical platform, and data analysis. Especially, automation of peak detection is an important issue for further development.

In the present study, we analyzed a well-known perturbation and successfully detected the metabolic pathway known to be affected in a single measurement. However, multiple measurements or supplementary techniques will be required for the analysis perturbations with unknown effects to accurately and precisely detect candidate pathways. Since our approach may suggest ideas contrary to common knowledge, we recommend that conclusions be verified with established techniques.

## 3. Experimental Section

### 3.1. Reagents

Yeast extract, peptone, and a yeast nitrogen base without amino acid were purchased from BD (Franklin Lakes, NJ, USA). d-glucose was purchased from Nacalai Tesque (Kyoto, Japan). ^13^C_6_-d-glucose was purchased from Cambridge Isotope Laboratory (Cambridge, MA, USA). For metabolite extraction and analysis, HPLC-grade methanol, HPLC-grade chloroform, HPLC-grade distilled water, and pyridine were purchased from Wako (Osaka, Japan). Methoxyamine hydrochloride was purchased from Sigma-Aldrich (St. Louis, MO, USA). *N*-methyl-*N*-(trimethylsilyl)trifluoroacetamide (MSATFA) was purchased from GL Sciences (Tokyo, Japan).

### 3.2. Yeast Cultivation

*Saccharomyces cerevisiae* BY4742 (*MATα*, *his3Δ 1*, *leu2Δ 0*, *lys2Δ 0*, *ura3Δ 0*) and reference strain X2180 (*MAT a/α*, *SUC2*, *mal*, *mel*, *gal2*, *CUP1*) were used for the experiment. The strain from the glycerol stock was streaked onto a yeast extract peptone dextrose (YPD) agar plate (10 g/L yeast extract, 20 g/L peptone, and 20 g/L glucose) to obtain a single colony isolate. For metabolic turnover analysis, cells from a single colony were picked and pre-cultured by inoculation onto 5 mL of synthetic defined (SD) medium (6.7 g/L yeast nitrogen base without amino acid, 20 g/L d-glucose) with or without amino acid supplement (80 mg/L histidine, 400 mg/L leucine, 80 mg/L lysine, and 80 mg/L uracil) and incubated overnight at 30 °C. The culture was then inoculated to 50 mL of fresh medium of the same composition as the pre-culture to an OD_590_ of 0.01 and grown at 30 °C in a rotary shaker (200 rpm) to an OD_590_ of 1.5. For the 0 s time point, 5 mL of culture was collected using a plastic syringe and syringe filter (25 mm diameter, 0.45 µm pore size, GL Sciences, Tokyo, Japan). The syringe filter was immediately soaked in liquid nitrogen to quench metabolism. The other cells were harvested rapidly by vacuum filtration using a polytetrafluoroethylene (PTFE) membrane filter (47 mm diameter, 1 µm pore size, Millipore, MA, USA). The filter was immediately placed onto 50 mL of fresh medium to re-suspend the cells. The medium composition was the same as the pre-culture except that d-glucose was replaced with 10 g/L U-^13^C_6_-d-glucose. The cells were incubated at 30 °C, and 5 mL of culture was collected at 10, 20, 40, 80, 160, 320, 640, 1280, and 2560 s after the suspension point using a syringe filter with the procedure described above. The samples were freeze-dried and stored at −80 °C until extraction.

For reference samples of ^12^C-peaks and ^13^C-peaks, cells of the X2180 strain from a single colony were inoculated to 5 mL of SD medium containing ^12^C-glucose or ^13^C-glucose and incubated overnight at 30 °C as a pre-culture. The culture was inoculated to 50 mL of fresh medium of the same composition as the pre-culture to an OD_590_ of 0.01 and grown at 30 °C in a rotary shaker (200 rpm) to an OD_590_ of 1.5. The cells were then collected by centrifugation (10,000× *g*, 4 °C, 5 min), freeze-dried, and stored at −80 °C until extraction.

### 3.3. Metabolite Extraction

For metabolic turnover analysis, 1 mL of mixed solvent (chloroform:methanol:water = 2 : 5 : 2) was applied to a syringe filter containing the yeast cells. The filtrate was collected and reapplied to the same filter. This procedure was performed two times. Then, 500 μL of distilled water was added to the filtrate and the mixture was vortexed. The sample was centrifuged (10,000 × *g*, 4 °C, 5 min), and the supernatant was collected in a 1.5-mL plastic tube. The sample was centrifugally dried and freeze-dried. The pellet was stored at −80 °C until GC/MS analysis.

### 3.4. Metabolites Derivatization

The sample was derivatized by oximation and silylation reagents. For oximation, 50 μL of methoxyamine hydrochloride in pyridine (20 mg/mL) was added before incubation at 30 °C for 90 min. For trimethyl silylation, 50 μL of MSTFA was added before incubation at 37 °C for 30 min.

### 3.5. GC/MS Analysis

GC/MS analysis was performed using a GC-2010 Plus gas chromatograph (Shimadzu) with an AOC-20is series injector/autosampler (Shimadzu, Kyoto, Japan) and a GCMS-QP2010 Ultra mass spectrometer (Shimadzu). A 30 m long × 0.25 mm inter diameter fused silica capillary column coated with 0.25 μm CP-SIL 8 CB low bleed/MS (Agilent Technologies, Santa Clara, CA, USA) was used. The front inlet temperature was 230 °C. The helium gas flow rate through the column was 1.12 mL/min. The column temperature was held isothermally at 80 °C for 2 min and then ramped from 80 to 330 °C by 15 °C/min and held isothermally for 6 min. The transfer line and ion-source temperatures were 250 and 200 °C, respectively. Twenty scans per second were recorded over the mass range 85–500 *m*/*z*.

### 3.6. Data Analysis

For metabolic turnover analysis, peaks and fragment ions labeled by carbons were manually selected by comparing data from non-labeled and ^13^C-labeled reference samples using GCMSsolution 4.11 (Shimadzu). The mass spectra of co-eluting peaks in both references were compared. The peaks were assigned as carbon containing peaks when the topologies of these two spectra were similar with higher *m*/*z* of some fragments in the ^13^C-labeled sample (e.g., Figure 4b). The shifted fragments were assigned as carbon-containing fragments. When the *m*/*z* of multiple fragments were shifted in a peak, the highest fragment and fragments that did not co-elute were used for further analysis. For peak area determination, the baseline of a fragment was set based on the most visible isotopomer. Peaks without visible isotopomers were excluded from turnover analysis. Negative peak areas were treated as zero. The isotopomer ratio (*IR*) was calculated using Equation (1). The data were standardized by the maximum value of each metabolite.

MetAlign [36] and AIoutput [23] were used for automatic metabolite identification. The identified peaks were also verified manually by comparing the spectra of samples to the library. Only the data from non-labeled samples were used for peak identification.

SIMCA 13 (UMETRICS, Umeå, Sweden) was used for principal component analysis (PCA). For differential analysis, values were calculated using Equation (2).

## 4. Conclusions

To achieve an unbiased understanding of metabolic dynamics, a non-targeted approach to isotope-assisted metabolomics is necessary. Time-resolved monitoring of isotopomers is essential for the analysis of global metabolism because the isotope labeling speed differs in a metabolite-dependent manner. Herein, we developed a framework for a non-targeted approach to metabolic turnover analysis using GC/MS. The combination of metabolic turnover analysis and multivariate differential analysis efficiently visualized the important perturbation-related peaks. By combining isotope and turnover data, metabolites were informatively annotated. In the present study, we focused on GC/MS analysis; however, this approach can be applied to the other analytical platforms such as liquid chromatography-MS and capillary electrophoresis-MS. We expect that this approach will provide new insight into perturbation-related metabolism, especially for chemicals, genes, and diseases that are biologically not well understood.

## Supplementary Files

Supplementary File 1
