# Supplementary material for: Novel Strategy for Non-Targeted Isotope-Assisted Metabolomics by Means of Metabolic Turnover and Multivariate Analysis
Source: Metabolites. 2014 Aug 25;4(3):722–39. doi: 10.3390/metabo4030722 (PMC4192689; doi:10.3390/metabo4030722)

Supplementary Information

Table S1. The loading (*p*) and the factor loading (*p*(*corr*)) of PCA in Figure 3.

Peak No.	Metabolite	p[PC1]	p[PC2]	p(corr) [PC1]	p(corr) [PC2]	*p*-Value [PC1]	*p*-Value [PC2]
Significantly correlated metabolites to PC1and PC2 after Bonferroni correction							
Peak-63	-	0.244	0.423	0.599	0.641	4.67×10^{-4}	1.34×10^{-4}
Significantly correlated metabolites to PC1 after Bonferroni correction							
Peak-30	-	0.562	−0.479	0.858	−0.451	1.41×10^{-9}	1.24×10^{-2}
Peak-08	Isoleucine_2TMS	0.364	0.185	0.835	0.262	9.68×10^{-9}	1.62×10^{-1}
Peak-48	Citric acid + Isocitric acid	0.321	−0.019	0.829	−0.031	1.48×10^{-8}	8.73×10^{-1}
Peak-57	Histidine	0.269	0.303	0.735	0.512	3.79×10^{-6}	3.85×10^{-3}
Peak-06	Valine_2TMS_Major	0.147	−0.073	0.884	−0.271	9.17×10^{-11}	1.47×10^{-1}
Peak-20	Malic acid	0.126	−0.031	0.697	−0.107	1.86×10^{-5}	5.74×10^{-1}
Peak-10	Proline	0.109	0.093	0.733	0.387	4.08×10^{-6}	3.45×10^{-2}
Peak-23	Aspartic acid	0.047	−0.046	0.699	−0.424	1.74×10^{-5}	1.94×10^{-2}
Peak-14	Serine_3TMS	0.041	−0.022	0.710	−0.237	1.13×10^{-5}	2.08×10^{-1}
Peak-61	-	−0.004	0.000	−0.650	0.001	1.01×10^{-4}	9.94×10^{-1}
Peak-54	Glucose	−0.005	0.000	−0.734	0.044	3.98×10^{-6}	8.16×10^{-1}
Peak-15	Alanine_3TMS	−0.032	0.014	−0.588	0.154	6.27×10^{-4}	4.15×10^{-1}
Peak-03	Alanine_2TMS	−0.037	0.025	−0.650	0.274	1.01×10^{-4}	1.43×10^{-1}
Peak-69	-	−0.054	0.007	−0.679	0.057	3.75×10^{-5}	7.64×10^{-1}
Peak-01	-	−0.083	−0.026	−0.591	−0.115	5.84×10^{-4}	5.47×10^{-1}
Peak-38	Asparagine	−0.111	−0.055	−0.667	−0.206	5.78×10^{-5}	2.74×10^{-1}
Peak-50	-	−0.139	0.203	−0.625	0.564	2.21×10^{-4}	1.16×10^{-3}
Significantly correlated metabolites to PC2 after Bonferroni correction							
Peak-55	Lysine_4TMS	0.069	0.230	0.367	0.760	4.61×10^{-2}	1.12×10^{-6}
Peak-51	Lysine_3TMS	0.091	0.217	0.453	0.670	1.20×10^{-2}	5.11×10^{-5}
Peak-49	Ornithine	0.033	0.092	0.468	0.810	9.18×10^{-3}	5.96×10^{-8}
Peak-16	Threonine_3TMS	0.003	0.087	0.035	0.734	8.56×10^{-1}	3.97×10^{-6}

Table S2. Spectral search result of Peak-30 in GMD.

Spectrum Linked to Analyte	RI of Spectrum	RI Difference	(1-Dotprod) Distance	Euclidean Distance	Hamming Distance	Jaccard Distance	s12gowleg Distance
Glutaric acid, 2-oxo- (1MEOX) (2TMS) MP	1572.85	8.05	0.2209	0.04	180	0.64	0.67
Malic acid, 2-isopropyl-(3TMS)	1570.38	5.58	0.4401	0.05	212	0.63	0.68
Filtering threshold of each parameter		10	0.5	0.5	500	0.9	0.9

Table S3. Predicted substructures of Peak-30 by decision tree on GMD.

Functional Group	Prediction	Probability*	Adjusted Probability	Support	Description	2-Isopropyl Malate
Hydroxy	present	98.56%	0.79%	828	intensity lg—191 ≥ 1.480192852 and intensity lg—217 ≥ 0.8222090721 and intensity ...	present
Carboxylic Acid Deriv	present	98.23%	1.41%	56	intensity lg—217 < 2.4857826471 and intensity lg—197 < 0.4003702641 and intensity ...	present
Alcohol	present	94.77%	1.55%	166	intensity lg—217 ≥ 1.7413686275 and < 2.3706843138 and mass difference—102 ≥ ...	present
Amine	absent	99.98%	0.05%	182	intensity lg—100 ≥ 1.5953158379 and < 2.0635438919 and intensity lg—89 ≥ 2.1427367687	absent
Acetal	absent	99.97%	0.00%	3484	intensity lg—361 < 0.5611459732 and intensity lg—204 < 2.3971363306	absent
Sec Alcohol	absent	99.94%	0.98%	64	intensity lg—217 ≥ 1.1120529413 and < 2.3706843138 and intensity lg—189 < 1.1370667458 ...	absent
1 2 Diol	absent	99.90%	0.43%	116	intensity lg—217 ≥ 1.1120529413 and < 2.3706843138 and intensity lg—146 ≥ 1.4999687433	absent
Phosphoric Acid Deriv	absent	99.84%	0.00%	3332	intensity lg—299 < 1.7852429867	absent
Prim Amine	absent	99.46%	0.03%	377	intensity lg—100 ≥1.547066617 and < 2.0313777447 and intensity lg—89 ≥ 2.1427367687	absent
Phenol	absent	99.12%	0.00%	2218	intensity lg—179 < 0.5885453224 and intensity lg—112 ≥ −0.109554863	absent
Aromatic	absent	99.10%	0.03%	1218	intensity lg—179 < 0.5885453224 and intensity lg—78 < 0.9362188339 and intensity ...	absent
Prim Aliph Amine	absent	99.06%	0.02%	216	intensity lg—100 ≥ 1.5953158379 and < 2.0635438919 and intensity lg—89 ≥ 1.7141051531	absent
Aldehyde	absent	99.03%	0.00%	1844	intensity lg—160 < 0.4984141588	absent
Alpha Aminoacid	absent	98.47%	0.00%	1558	intensity lg—89 ≥ 1.7141051531	absent
alphaAminoAcid 2TMS	absent	98.30%	0.00%	679	intensity lg—100 < 1.9145779788	absent
Prim Alcohol	absent	98.18%	0.11%	1784	intensity lg—103 < 1.6016308665 and intensity lg—205 < 1.8810598373	absent
Amine 2TMS	absent	97.25%	0.00%	902	intensity lg—174 < 2.4918380737	absent

Table 3. *Cont.*

Functional Group	Prediction	Probability*	Adjusted Probability	Support	Description	2-Isopropyl Malate
Carboxylic Acid Ester	absent	94.73%	0.00%	1386	intensity lg—87 < 2.5117663383 and intensity lg—239 < 1.8445128918 and intensity ...	absent
Heterocycle	absent	90.30%	0.02%	121	intensity lg—361 < 1.1900148869 and intensity lg—117 < 2.5344496608 and intensity ...	absent

* filtering threshold was 90%.

Table S4. Detected substructures of Peak-63 by decision tree on GMD.

Functional Group	Prediction	Probability *	Adjusted Probability	Support	Description	2-Oxoglutarate
Alcohol	present	97.43%	2.79%	190	intensity lg—103 ≥ 2.067753911 and < 2.5338769555 and intensity lg—217 ≥ 1.7413686275 ...	absent
Amine	present	96.02%	17.52%	267	intensity lg—100 ≥ 2.0635438919 and intensity lg—89 < 1.7141051531 and intensity ...	present
Carboxylic Acid Deriv	present	95.99%	1.06%	72	intensity lg—217 < 2.3706843138 and RI var5 < 2633.8660400391 and intensity lg ...	present
Prim Amine	present	95.33%	20.54%	205	intensity lg—100 ≥ 2.2174140453 and intensity lg—130 ≥ 1.1838381767 and intensity ...	present
Prim Aliph Amine	present	93.98%	24.10%	422	intensity lg—100 ≥ 2.0313777447 and intensity lg—99 < 1.4552190542 and intensity ...	present
Aromatic	present	91.14%	15.82%	31	intensity lg—179 < 0.5885453224 and intensity lg—78 < 0.4202735424 and intensity ...	present
Acetal	absent	99.97%	0.00%	3484	intensity lg—361 < 0.5611459732 and intensity lg—204 < 2.3971363306	absent
1 2 Diol	absent	99.90%	0.43%	116	intensity lg—217 ≥ 1.1120529413 and < 2.3706843138 and intensity lg—146 ≥ 1.4999687433	absent
Phosphoric Acid Deriv	absent	99.84%	0.00%	3332	intensity lg—299 < 1.7852429867	absent
Alkene	absent	99.55%	0.00%	934	intensity lg—123 < 0.5563361645 and intensity lg—100 ≥ 1.1270877838 and intensity ...	absent
Phenol	absent	99.53%	0.00%	1756	intensity lg—179 < 0.8386740685 and intensity lg—324 < 0.7822675705 and intensity ...	absent

Table 4. *Cont.*

Functional Group	Prediction	Probability *	Adjusted Probability	Support	Description	2-Oxoglutarate
Carboxylic Acid Ester	absent	98.91%	0.00%	1728	intensity lg—87 < 2.5117663383 and intensity lg—239 < 1.8445128918 and intensity ...	absent
Aldehyde	absent	98.70%	0.00%	1086	intensity lg—160 ≥ 0.4984141588 and < 1.4990484953 and intensity lg—206 < 1.3138294458	absent
Carbonyl	absent	98.50%	0.03%	1625	intensity lg—89 < 1.2854735374 and RI var5 < 2633.8660400391	absent
alphaAminoAcid 2TMS	absent	97.83%	0.00%	154	intensity lg—100 ≥ 1.9145779788 and intensity lg—218 < 1.2007082462	absent
Amine 2TMS	absent	97.25%	0.00%	902	intensity lg—174 < 2.4918380737	absent
Prim Alcohol	absent	96.82%	0.17%	185	intensity lg—103 ≥ 1.7246618986 and < 2.1497745991 and intensity lg—105 ≥ 1.2191258907	absent
Alpha Aminoacid	absent	95.36%	0.00%	144	intensity lg—89 < 1.2854735374 and intensity lg—128 < 1.3681855202 and RI var5 ...	present

* filtering threshold was 90%.

Figure S1. Mass spectrum of Peak-63. (**a**) Mass spectra of Peak-63 in the sample (**upper**) and Histidine-4TMS in Golm Metabolome Database (**lower**); (**b**) Mass spectra of the ^{12}C (**upper**) and ^{13}C (**lower**) monoisotopic mass of Peak-63; (**c**) The magnified figure of mass spectra surrounded by dotted line.

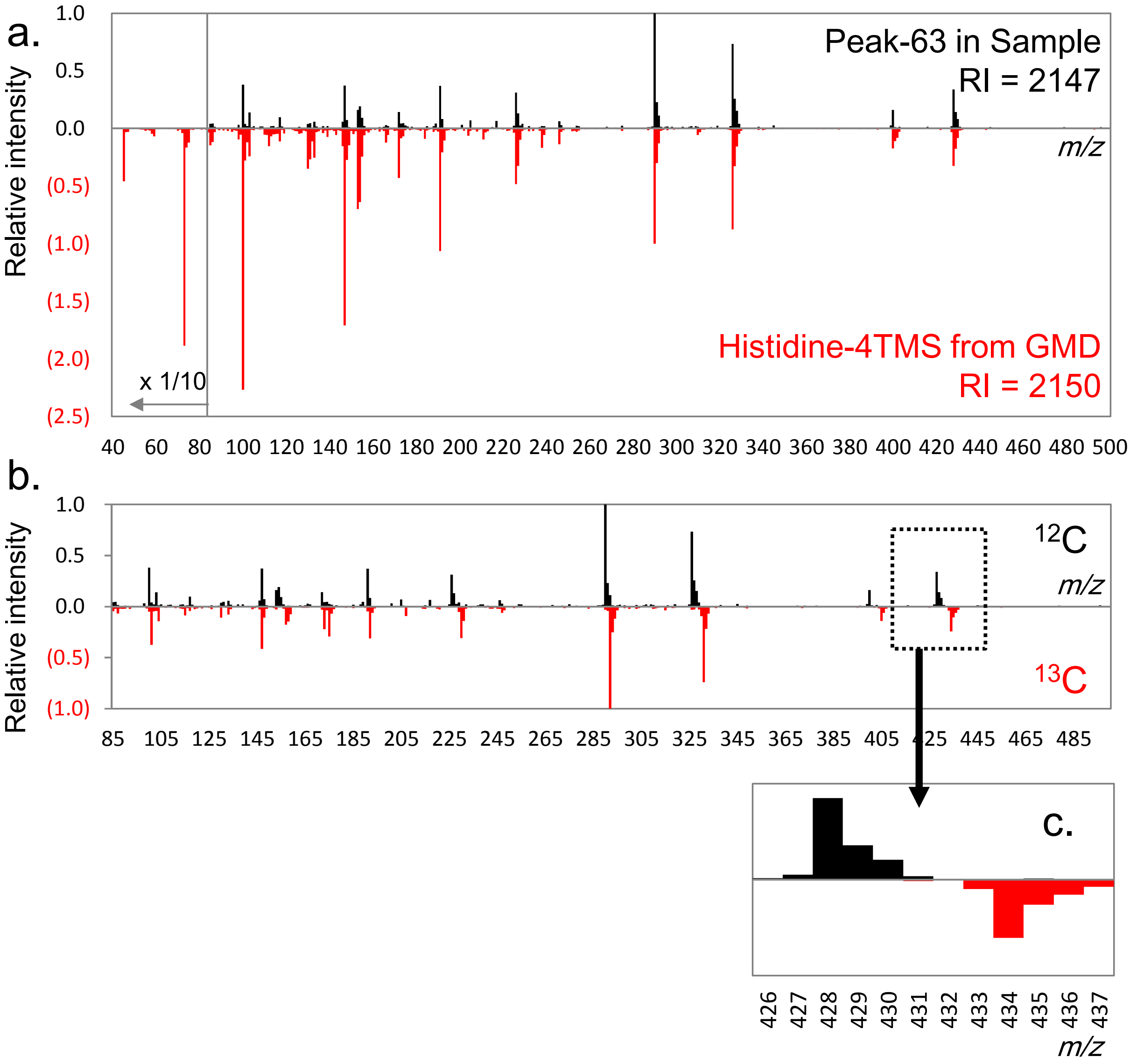

Figure S2. Spike experiment of standards to *S.cerevisiae* extracts. Chromatograms and mass spectra of 100 nmol of 2-isopropyl malate (2IP), 100 nmol of histidine (His), *S.cerevisiae* extract of approximately 1 mg dry cell weight (Yeast) and their mixture (2-IP:His:Yeast = 1:1:2). Procedures of derivatization and GC/MS analysis was same in the text. (**a**) Left figure shows the chromatograms around 9.54 min of retention time (RT). Right figures are corresponding mass spectra of peaks at 1566 of retention index (RI), which are indicated by the black arrow in the left figure; (**b**) Left figure shows the chromatograms around 13.60 min of retention time (RT). Right figures are corresponding mass spectra of the peak at 2147 of retention index (RI), which are indicated by black arrow in the left figure. Although the peak was not detected in the pure histidine standard, it was increased in the spiked sample.

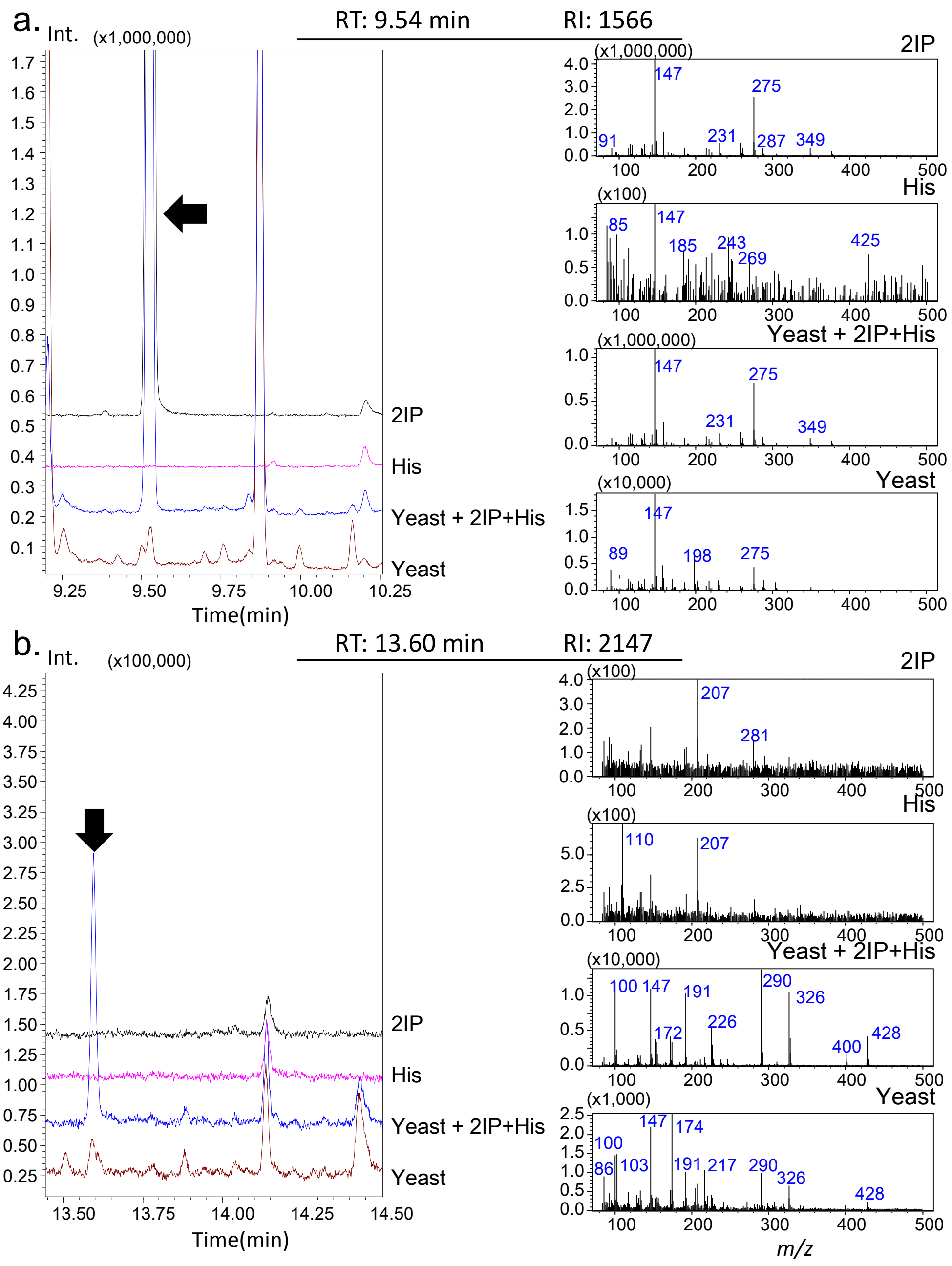

Supplement: Supplementary File 1 [file metabolites-04-00722-s001.pdf]
